# Vasoplegic Syndrome Following Bypass: A Comprehensive Review of Pathophysiology and Proposed Treatments

**DOI:** 10.7759/cureus.78057

**Published:** 2025-01-27

**Authors:** Jaime P Torrez, Denise A Otsuki, Suely P Zeferino, Ana F Sanchez, José Otávio C Auler Jr

**Affiliations:** 1 Anesthesiology, Faculdade de Medicina da Universidade de São Paulo, São Paulo, BRA; 2 Anesthesiology, Hospital das Clínicas da Faculdade de Medicina da Universidade de São Paulo, São Paulo, BRA; 3 Anesthesiology, Hospital das Clinicas da Faculdade de Medicina da Universidade de São Paulo, São Paulo, BRA

**Keywords:** bypass, clinical predictors, hydroxocobalamin, methylene blue, treatment, vasoplegic syndrome

## Abstract

Following cardiopulmonary bypass (CPB) surgery, patients may experience vasoplegic or vasogenic shock syndrome. This condition has a variable incidence, reaching up to 44% in high-risk patients, with mortality rates ranging from 30% to 50%, primarily due to multiple organ failure. This complex condition is characterized by low arterial pressure, unresponsive vascular collapse to high doses of vasopressors, and biochemical signals of cellular oxygen debt. The cardiac output can either be low or abnormally elevated. A fundamental aspect of the pathophysiology of vasogenic syndrome after CPB is related to the dysfunction of vascular smooth muscle cell contraction. This syndrome is often associated with complex cardiac surgery such as reoperations, long periods of bypass and aorta clamping, and excessive blood transfusion. Some potential triggers that might lead to this condition include the preoperative use of antagonists of the renin-angiotensin system, calcium blockers antagonists, and chronic renal disease. Recent literature has advocated treating vasoplegic syndrome after bypass using oxide nitric synthase inhibitors, such as methylene blue and hydroxocobalamin, along with the progressive escalation of potent vasopressors and intravascular volume adjustment.

## Introduction and background

Surgical injury is well known to produce inflammatory stimulus, which may lead to an immune response that can contribute to postoperative morbidity and mortality. Most of this response is translated by a circulatory hyperdynamic reaction, including low systemic resistance and transient hypotension. This response contains neurohumoral and inflammatory-immune elements, primarily determined by the surgical injury’s magnitude and duration and the comorbidities’ presence [1].

When the circulatory picture is extremely severe, characterized by normal or increased cardiac output, low systemic vascular resistance (SVR), and vasopressor refractoriness, it is called “vasoplegic syndrome.” This condition is commonly observed in septic shock, post-cardiac bypass, after trauma, major surgery, and severe burns. After bypass surgery, the reported incidence varies from 5% to 20%, mainly depending on the previous clinical situation, the length of surgery, and the duration of bypass surgery [2].

In cardiac surgery, inflammation may be exacerbated by the bypass procedure and can present in varying degrees. During cardiopulmonary bypass (CPB), hypotension frequently occurs due to acute hemodilution, typically corrected by adjusting pump flow and administering lower doses of vasopressors. In contrast, vasoplegia, or vasoplegic shock, characterized by profound hypotension refractory to vasopressors, may emerge during attempts to wean off bypass or in the hours following the procedure, even in the operating room or intensive care unit. In such cases, arterial pressure may not be maintained adequately despite higher vasopressor doses [2]. This clinical scenario of circulatory collapse necessitates differential diagnoses of cardiac dysfunction, which can be evaluated by directly measuring cardiac output using pulmonary catheters or echocardiography [3].

The pathophysiology of vasoplegia is complex and not fully understood but involves many factors; once CPB is initiated, the blood in contact with non-endothelial surfaces starts an extensive endothelial dysfunction with the release of inflammatory mediators such as tumor necrosis factor alpha and IL-1. These mediators bind to endothelial receptors and activate the endothelial cells. Otherwise, nitric oxide (NO) dysregulation, vasopressin depletion, abnormal hydrogen sulfide metabolism, and prostaglandin release may be part of this process, discussed in detail in the following section. Vasoplegic syndrome may also be amplified by ischemia-reperfusion syndrome in several organs caused by an aortic cross-clamp and de-clamping [4].

The literature on vasoplegic syndrome after bypass has seen a growth in the number of publications. Reports dating back to the 1980s by Kirklin refer to damage following bypass, which was then known as postperfusion syndrome [5]. Gomes et al. reported six cases in which they defined vasoplegic syndrome following open-heart surgery as characterized by severe hypotension, normal or elevated cardiac output, decreased atrial filling pressure, and low SVR [6].

## Review

Definition

**Figure 1:**
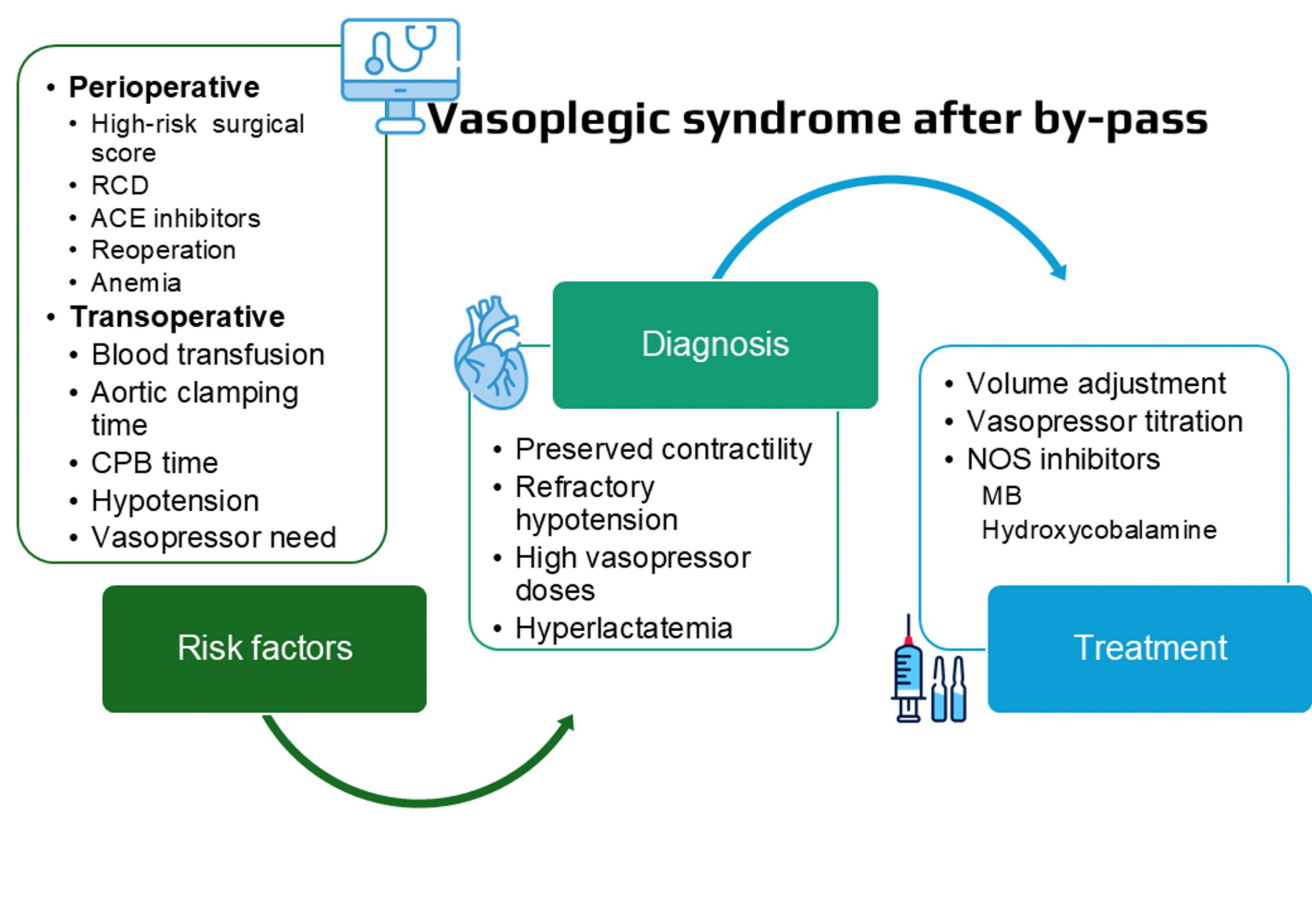
Key points of vasoplegic syndrome after CPB ACE, angiotensin-converting enzyme; CPB, cardiopulmonary bypass; MB, methylene blue; NOS, nitric oxide synthase; RCD, renal chronic disease

The vasoplegic syndrome after bypass cardiac surgery has been defined in various ways over time. Most definitions are based on the variable cutoff of mean arterial pressure (MAP). The more common definition is when the MAP drops below 50 mmHg and requires a norepinephrine dosage of more than 0.08 mcg/kg/min or when there is hypotension with low SVR of <800 dynes/s/cm and preserved cardiac index (CI) >2.2L /min/m² [7]. It is important to know the clinical implications of vasoplegia, categorized as mild (MAP 50-60 mmHg and requiring one vasopressor), moderate (MAP 50-60 mmHg and requiring two or more vasopressors or MAP <50 mmHg and administration of one vasopressor), or severe (MAP <50 mmHg, and requiring administration of two or more vasopressors). The last one is associated with a worse outcome (Figure 1) [2,7].

Clinical risk factors

Cardiothoracic surgical teams focus on identifying risk factors associated with cardiopulmonary vasoplegic syndrome before or during surgery to prevent or reduce its negative impact. Although several publications have been made on this topic, most are either retrospective or based on small case studies and are not robust enough. A meta-analysis by Dayan et al. summarized these risk factors. The total number of patients included was 30,035 patients (1,524 with vasoplegia and 28,511 without vasoplegia). The only preoperative variable that was significantly associated with pvasoplegia (PV) was renal failure (OR 1.47; 95% CI 1.17-1.86). Patients with isolated coronary artery bypass grafting had a lower risk for PV (OR 0.36; 95% CI 0.22-0.59), whereas previous cardiac surgery (OR 2.03; 95% CI 1.27-3.26) and combined procedures increased its incidence (OR 2.12; 95% CI 1.82-2.47). PV increased with higher use of red blood cells (OR 2.12; 95% CI 1.82-2.47), longer aortic cross-clamp (mean difference 8.15 min; 95% CI 8.79-27.50 min), and CPB (mean difference 25.72 min; 95% CI 12.75-38.69 min) duration [8].

Although most preoperative clinical factors identified by different studies coincide, there is some controversy around certain factors.

Argenziano et al. conducted a prospective study to determine the incidence and clinical predictors of post-bypass vasoplegia and its correlation with plasma arginine vasopressin levels (AVP). Among the patients with post-CPB vasodilatory shock (8% incidence), most had AVP levels that, although within the normal osmoregulatory range for healthy normotensive subjects, were inappropriately low for the present degree of arterial hypotension. This result suggests that AVP deficiency contributes to the development of vasodilatory shock. They also found that angiotensin-converting enzyme (ACE) inhibitor therapy is an independent contributor to the development of vasoplegic syndrome after bypass surgery [9]. Additionally, Colson et al. have found that patients who presented with post-cardiac surgery vasoplegia had higher pre-CPB copeptin levels, a marker of vasopressin release [10].

Mekontso-Dessap et al. conducted a study on 32 patients who experienced vasoplegic syndrome after coronary artery bypass surgery. The study revealed that two factors were independent predictors of vasoplegia after bypass surgery - preoperative use of ACE inhibitors (relative risk 2.26) and intravenous heparin (relative risk 2.78), based on multivariate analysis [11].

Levin et al. conducted a retrospective analysis of the medical records of 2,383 adult patients who underwent cardiac surgery. Out of these, 577 patients (20.4%) were clinically diagnosed with vasoplegic syndrome after CPB. Their study showed that 1,645 patients (58.3%) had a significant drop in MAP after starting CPB (decline in MAP >20% from pre-bypass baseline that lasted >2 min) and were significantly more likely to develop vasoplegia (23.0% versus 16.9%). Patients who experienced a significant drop in MAP were also more likely to have adverse postoperative outcomes [12].

Kortekaas et al. described that previous endothelium dysfunction could predict vasoplegia after bypass surgery. The concept is that during on-pump cardiac surgery, the endothelial cells are exposed to multiple aggressions, which decreases vasomotor tone and vascular systemic resistance, resulting in hypotension. Preexisting endothelial cell activation, reflected by higher baseline von Willebrand factor propeptide and sP-selectin levels, is a predisposing factor for post-cardiac surgery vasoplegia [13].

Other described risk factors were preoperative anemia and higher thyroxine levels in a retrospective study of 225 heart failure patients who underwent left ventricle wall correction and mechanical device implantation. According to the authors, these factors can guide treatment strategies [14].

In another study by van Vessem et al., 122 patients who underwent mitral valve replacement were investigated for the incidence of vasoplegia. The incidence of vasoplegia was 19%. Patients without vasoplegia after pump had a higher long-term survival rate (65% vs. 93%, p < 0.001). After adjusting the data for age, gender, and heart failure etiology, it was found that prior anemia (OR 3.00; 95% CI 1.10-8.20; p = 0.032), length of cross-clamp (OR 1.03; 95% CI 1.01-1.04; p = 0.001), CPB (OR 1.01; 95% CI 1.00-1.02; p = 0.003), and surgical time (OR 1.01; 95% CI 1.00-1.02, p = 0.002) increased the risk of vasoplegia [15].

Magoon et al. hypothesized the potential prognostic of inflammatory hematological indices, defined as platelet‐leucocyte interaction indices (PLIs) and leucocyte indices (LIs), in predicting vasoplegic syndrome after CPB. The hematological profile in 1,045 patients identified LIs and PLIs as predictors of vasoplegic syndrome, and demographic data such as diabetes, chronic renal disease, elderly, poor left ventricle ejection fraction, and higher EuroScore were also signalized as significant [16].

Tsiouris et al. conducted a retrospective analysis of the medical records of 1,992 patients who underwent cardiac surgery involving CPB. According to their analysis, 20.3% (n = 405) of the patients developed vasoplegia. The study identified several factors that increase the risk of developing vasoplegia. These factors include patients undergoing valve surgeries, heart transplants, having dialysis-dependent renal failure, being aged 65 or over, receiving diuretic therapy, and having experienced a recent myocardial infarction. Interestingly, the researchers found that preoperative beta-blocker agents were a protective factor against vasoplegia [2].

Various studies have identified several factors contributing to vasoplegia during CPB. These included advanced age (>65 years), low left ventricular ejection fraction (<40%), presence of left ventricular assistance device, prolonged CPB (>190 min), early hypotension after the initiation of the bypass pump, perioperative use of ACE inhibitors, obesity, and endocarditis. Considering these factors can help predict and prevent the likelihood of vasoplegic syndrome in surgical patients [17].

Furthermore, patients with an increased risk of vasoplegia could potentially benefit from additional preoperative measures such as optimization of the hemodynamic situation and renal function. In addition, in patients with an increased risk of vasoplegia, early initiation of a vasopressor regime (vasopressin or methylene blue (MB)) in the perioperative phase could be considered (Table 1).

**Table 1:** Risk factors for post-bypass vasoplegia ACE, angiotensin-converting enzyme; AVP, arginine vasopressin; CABG, coronary artery bypass graft; CPB, cardiopulmonary bypass; MAP, mean arterial pressure

Study	Design	Number of patients	Incidence	Risk factor
Tsiouris et al. (2017) [2]	Retrospective	1,992 cardiac surgeries on CPB	20.30%	Valve operations, heart transplants, dialysis-dependent renal failure, age >65 years, diuretic therapy, and recent myocardial infarction
Argenziano et al. (1998) [9]	Prospective	145 CPB	8%	Low ejection fraction, ACE inhibitor use, and low serum AVP levels
Mekontso-Dessap et al. (2001) [11]	Retrospective; case-control (36:72)	1,413 CABG patients with normal ventricular function	2.50%	ACE inhibitors and intravenous heparin
Levin et al. (2009) [12]	Retrospective	2,383 cardiac surgeries on CPB	20.40%	Drop in MAP after initiating CPB
Kortekaas et al. (2013) [13]	Prospective	40 mitral valve surgeries on CPB	38.50%	Preexisting endothelial cell activation, indicated by higher baseline von Willebrand factor propeptide and sP-selectin levels
van Vessem et al. (2017) [14]	Retrospective	225 surgical left ventricular restoration	29%	Anemia and elevated thyroxine levels
van Vessem et al. (2019) [15]	Retrospective	122 patients undergoing mitral valve replacement	19%	Anemia and prolonged procedure times
Magoon et al. (2022) [16]	Retrospective	1,045 cardiac surgeries on CPB	19.60%	Platelet-leukocyte indices

Pathophysiology 

**Figure 2:**
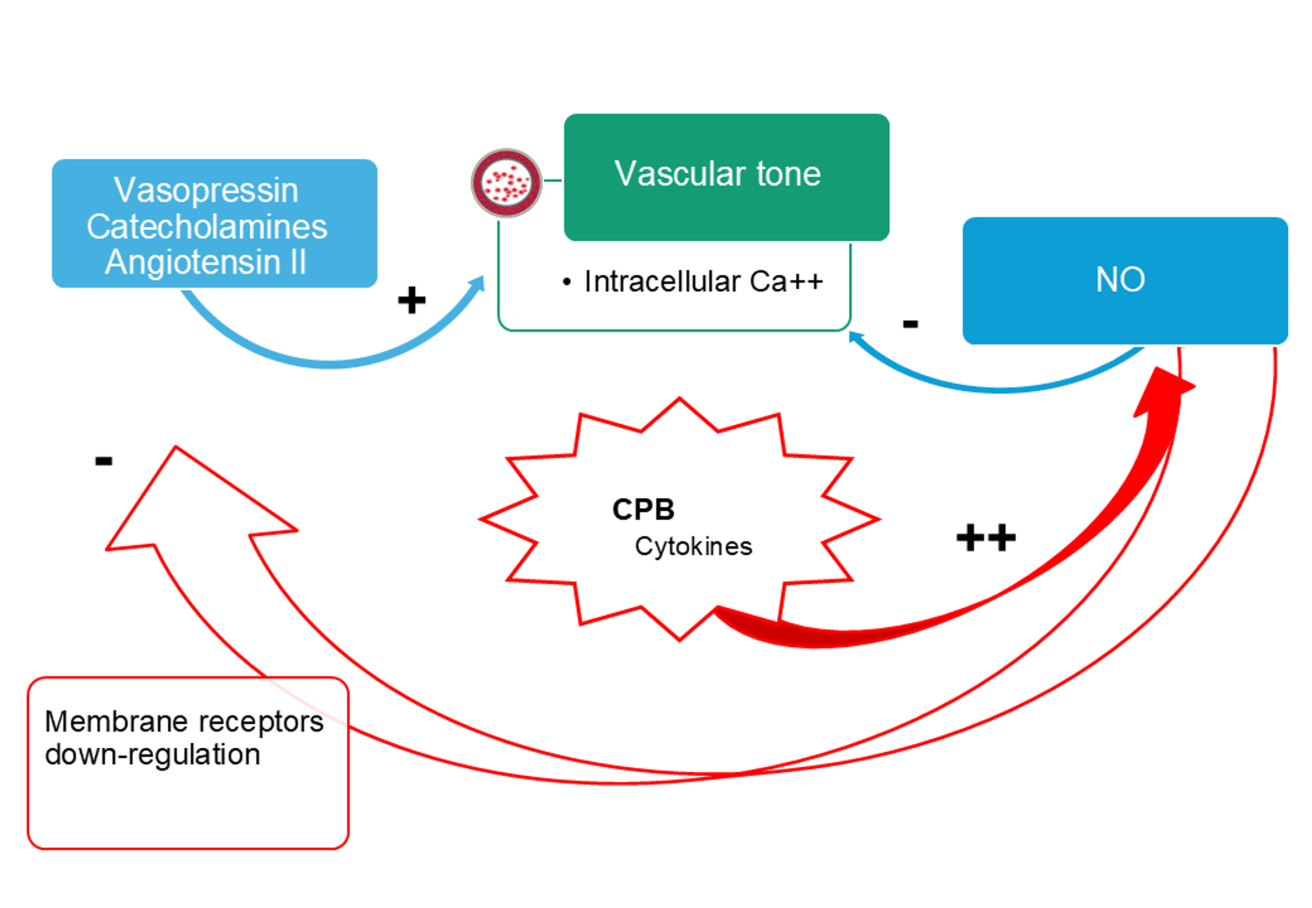
Components of vascular tone

Vasoplegic syndrome can occur during and after bypass surgery, and its severity depends on several factors, including patient characteristics and the type of surgical procedure as described before. The syndrome is primarily caused by a dysfunction in the mechanisms responsible for contracting and relaxing smooth muscle cells (Figure 2). In severe cases, it can mimic circulatory shock and be accompanied by signals such as hypotension, metabolic acidosis, hyperlactatemia, and resistance to vasopressors and volume expansion; however, cardiac output remains normal in most cases or slightly elevated [18].

Since this syndrome is complex and triggered by several factors, we have divided this section into topics to facilitate better understanding.

Normal Physiology of Contraction and Relaxation of Smooth Muscle Cells

Vascular tone, or the contractile state of vascular smooth muscle, is regulated by intracellular calcium levels, which control myosin light chain (MLC) phosphorylation necessary for muscle contraction. Contraction occurs when calcium is released from the sarcoplasmic reticulum and enters the cell through voltage-sensitive channels, leading to myosin-actin crosslinking. Relaxation happens when calcium levels decrease due to calcium uptake and potassium efflux, resulting in membrane hyperpolarization and vasodilation [18].

Vascular tone is influenced by both intrinsic and extrinsic mechanisms. Intrinsic factors include the release of NO, endothelin, and prostacyclin by the endothelium. NO, which activates soluble guanylyl cyclase to produce cGMP, triggers calcium efflux and smooth muscle relaxation. This process is regulated by three types of NO synthases (NOSs): nNOS (neuronal), eNOS (endothelial), and iNOS (inducible). Inflammatory conditions can activate iNOS, leading to excessive NO release and prolonged vasodilation and hypotension [3].

Extrinsically, vascular tone is controlled by sympathetic nervous activity and neurohormones like vasopressin, angiotensin II, and catecholamines. Catecholamines bind to G protein-coupled receptors, triggering calcium release and vasoconstriction. In conditions like vasoplegia, receptor phosphorylation and excessive NO production can reduce norepinephrine responsiveness, contributing to poor response to catecholamines, further exacerbated by low vasopressin levels, acidosis, and membrane hyperpolarization due to KATP channel activation [3,19].

Bypass Triggering Inflammation

Damage-associated molecular patterns trigger the inflammatory immune response to surgical injury, including bypass procedures, by binding to pattern recognition receptors. This activation leads to the release of proinflammatory cytokines, chemokines, and other molecules, driving inflammation based on clinical factors and genetic predisposition. Key cytokines in this process include IL-1 (which causes fever, mediator release, and endothelial damage), IL-6 (which stimulates corticosteroid and ACTH production), IL-8 (a neutrophil chemotactic agent), and IL-10 (an anti-inflammatory cytokine that suppresses NF-kB). An imbalance between proinflammatory and anti-inflammatory factors can lead to vasoplegic syndrome, characterized by increased NO production, adrenoreceptor desensitization, and deficiencies in vasopressin and prostanoids [20].

Predicting excessive inflammatory responses in patients is challenging, but factors like leukocytosis and younger age may help. Genetic variations, such as IL-6 gene polymorphisms, also explain differences in patient response. In diabetic patients, upregulation of genes like MYC/JUN and cytokines IL-1β and IL-8, along with increased vascular endothelial growth factor (VEGF) expression, has been observed. Elevated circulating VEGF levels are associated with greater weight gain and longer hospital stays due to fluid leakage from the capillaries [21].

Cytokine release from non-endothelial surfaces activates iNOS, leading to prolonged NO production. NO induces vasodilation in smooth muscle cells through activation of soluble guanylyl cyclase, which generates cGMP, leading to MLC dephosphorylation, potassium efflux, and membrane hyperpolarization. This reduces cytosolic calcium and promotes vasodilation, further enhanced by NO-cGMP activation of KCa++ channels. NO also forms peroxynitrite, which further dilates blood vessels [22].

Vasopressin, a neurohormone, typically causes vasoconstriction by binding to the AVPR1a receptor. However, during bypass surgery, low vasopressin levels and increased NO production can cause vasodilation. Vasopressin treatment can help manage vasoplegia, a condition where blood vessels are excessively relaxed. Studies show that patients with vasoplegia post-surgery have lower vasopressin levels, possibly due to factors like elevated cardiac filling pressures or autonomic dysregulation [9].

Glycocalyx and endothelium: CPB can cause glycocalyx degradation, which is linked to the CPB-induced systemic inflammatory response. The glycocalyx, a protective layer on the endothelial wall, can be damaged, leading to inflammatory cell and fluid migration. Glycocalyx degradation is measured by plasma syndecan-1 levels. Studies have shown that inflammation, marked by proinflammatory cytokines (IL-6 and IL-8) and anti-inflammatory cytokine IL-10, is associated with glycocalyx damage. A study of 30 cardiac surgery patients found that increased IL-6, IL-8, and IL-10 correlated with glycocalyx degradation, but no link was found with clinical outcomes [23].

Stahl et al. suggested that complement activation can occur via spontaneous hydrolysis of the C3 complement fraction, with significant activation occurring when blood contacts CPB surfaces. The artificial materials in CPB circuits play a key role in early complement activation. Cd4 complexes, linked to arrhythmias post-CABG, serve as markers of delayed complement activation. The impact of complement activation on outcomes varies and may be influenced by genetic factors [24].

Coagulation disorders associated with inflammation: Coagulation disorders often occur with severe inflammation. Exposure to artificial materials during surgery triggers intrinsic coagulation via factor XIIa, activating factors XI and X. This, combined with extrinsic coagulation, forms the prothrombinase complex, converting prothrombin to thrombin. Thrombin, generated in large amounts, stimulates von Willebrand factor production and plasminogen, leading to fibrinolysis. It also affects neutrophil adhesion and promotes endothelial damage. Tissue factor (TF) on subendothelial cells activates factor X, while leukocyte TF exposure activates factor VII, contributing to consumptive coagulopathy, bleeding, thrombosis, and fibrinolysis [25].

Inflammation self-limitation: After complex surgery, inflammation may fluctuate but typically resolves within hours or days. Neutrophils and macrophages, derived from monocytes, initiate resolution, with prostaglandin E2 playing a key role in shifting neutrophil lipid mediator biosynthesis. This decreases proinflammatory leukotriene and NO production, signaling the end of acute inflammation [26].

Role of genomics

Functional genomics has enhanced our understanding of inflammation in cardiac bypass surgery. Despite improvements in surgical techniques, inflammation remains common and variable. Research is focusing on identifying genetic factors and biomarkers that influence inflammation outcomes. A study of 12,625 genes in CABG patients found specific gene expressions linked to inflammation, apoptosis, and oxidative stress. Variants of IL-6 (-572G) may contribute to inflammation variability post-surgery [27,28].

Treatment

In cardiac surgery, hypoxia, anemia, and inadequate tissue perfusion can occur due to reduced blood flow to the cells or unrecognized low cardiac output during periods not compensated by bypass flow. As a result, the primary goal of any surgery, especially in high-risk patients, is to prevent tissue hypoperfusion and ensure adequate oxygenation of the tissues. Additional factors such as aortic cross-clamping, hemodilution, and direct cardiac intervention can also impair oxygen delivery to tissues, potentially increasing lactate levels. Furthermore, the inflammatory response can lead to endothelial dysfunction, disrupting microcirculation and causing extravascular fluid losses and “false” hypovolemia. To address these concerns, it is essential to monitor cardiac output, assess contractility, evaluate cardiac function, and predict fluid responsiveness. Cellular oxygenation can be gauged through lactate measurement and the carbon dioxide gradient. In cases where a patient develops cardiovascular dysfunction and the anesthesiologist encounters hypotension unresponsive to conventional vasopressor doses, which is not related to intrinsic cardiac contractility, the vasoplegic syndrome may be a consideration [7].

The initial step is the optimization of fluid and cardiac status. When addressing insufficient oxygen delivery, the resuscitation goal is improving oxygen delivery. This can be accomplished by increasing cardiac output through volume loading, inotropic drugs, and potentially blood transfusions to raise hemoglobin levels. However, improperly administered fluid for treating under-perfused tissue can lead to a positive fluid balance, which is linked to longer ICU stays, extended hospital stays, and higher mortality rates [7,29,30].

Various monitoring methods in the operating room are available to identify preload responsiveness for guiding volume treatment. While these techniques have limitations, they are often used in combination. In cardiac surgery patients, pulse pressure and stroke volume variations are helpful when the chest is closed, or superior vena cava diameter variations can be assessed using transesophageal echocardiography. Both are practical tools for guiding fluid infusion to evaluate fluid responsiveness and direct fluid challenges [31].

After volume assessment and necessary correction, norepinephrine can be started if arterial pressure remains below 65 mmHg. Norepinephrine is the first choice, a potent and reliable vasopressor that can treat hypotension; the initial dose is 0.01 mcg/kg/min, which can be increased to the next 0.5-1.0 mcg/kg/min or more. The next step is to consider vasopressin, which has selective activity for the V1 receptor located on vascular muscle. Infusion usually starts at 0.01 to 0.06 U/min. Angiotensin II has been used at doses of 10-40 ng/kg/min as an effective therapy to raise blood pressure by binding to specific angiotensin receptors on the cell membrane of various cell types, with angiotensin II receptor type 1 being primarily responsible for vasoconstriction [7]. A meta-analysis of randomized controlled trials including 625 adult patients undergoing elective or emergency cardiac surgery was submitted to Interventions: AVP infusion (n = 313) or control/standard therapy (n = 312). In summary, the results showed that vasopressin may decrease the incidence of perioperative complications, but no difference in postoperative mortality was detected. It is recommended to associate potent vasopressors to avoid side effects from excessive individual doses that may cause severe ischemia in splanchnic organs, kidneys, and limb extremities. Vasopressin alone as a vasopressor to treat vasoplegic states is still a matter of controversy (Table 2) [32].

**Table 2:** Summary of current literature treatment recommendations

Vasoplegia treatment strategy	
Category	Treatment strategy
Optimize cardiac preload and output	Goal-directed therapy; fluids
Conventional vasopressors	Norepinephrine; vasopressin
Nonconventional vasopressors (rescue therapy)	Methylene blue; hydroxocobalamin

Pharmacological Treatment

The management of vasoplegia using pharmacological treatments is a topic of debate, and it can be categorized into two main types: preventive and direct treatments. Preventive agents may include corticosteroids, which inhibit proinflammatory cytokines, and vitamin C, which acts as a cofactor for catecholamine synthesis. Direct treatments identified in the literature include MB, which inhibits guanylyl cyclase, and hydroxocobalamin, used to inhibit the production of NO and hydrogen sulfide [33-35].

Corticosteroids: Corticosteroids have been suggested to mitigate inflammatory responses during major surgeries. These agents can lower serum inflammatory markers and interfere with the inflammatory cascade. However, the routine use of corticosteroids to prevent significant inflammatory reactions remains a subject of debate and may not offer substantial advantages, as pointed out by Chaney [36].

More recently, the question of corticosteroids to reduce inflammation in cardiac surgery is still open, but it is uncertain if they prevent major adverse events, including vasoplegic states. A randomized, controlled, double-blind group study was conducted to evaluate the effects of high-dose dexamethasone on significant adverse events during cardiac surgery. Patients were randomly assigned to receive a single intraoperative dose of 1 mg/kg dexamethasone (n = 2,239) or placebo (n = 2,255). The study found that dexamethasone reduced postoperative infection, duration of postoperative mechanical ventilation, and lengths of intensive care unit and hospital stays. However, it did not lessen the 30-day incidence of major adverse events compared to placebo. Nevertheless, dexamethasone was found to be associated with higher postoperative glucose levels. The authors did not report any comparison or different incidences between groups of vasoplegia [34].

In a recent clinical study, researchers aimed to determine whether the use of steroids in patients undergoing CPB during cardiac surgery could suppress inflammatory responses and improve outcomes for those at high risk of morbidity and mortality. The study was a double-blind, randomized, and controlled trial that involved 7,507 patients across 80 hospital or cardiac surgery centers in 18 countries. Each patient was randomly assigned to receive either methylprednisolone (total 500 mg) or a placebo. However, the results of the trial showed that the use of methylprednisolone did not significantly reduce the risk of death or significant morbidity following 30 days of cardiac surgery with CPB. The mortality rate was almost similar in both groups, with 6% of patients receiving methylprednisolone and 5% of patients receiving a placebo. Moreover, the risk of death or significant morbidity was also similar in both groups, which was around 24%. Expected outcomes in both groups included infection, surgical site infection, and delirium. Therefore, the authors concluded that the trial does not support the routine use of methylprednisolone for high-risk patients undergoing CPB during cardiac surgery [37].

Vitamin C: Vitamin C has immunomodulatory and antioxidant properties, making it a potential treatment for sepsis-induced hypotension and a survival rate. However, a recent trial of randomized-controlled septic patients combined high-dose intravenous vitamin C (1.5 g), thiamine 100 mg, and hydrocortisone every six hours versus placebo did not significantly increase ventilator and vasopressor-free within 30 days [38].

In another trial conducted in two tertiary ICUs, only a high vitamin C dose was administered. The authors defined vasoplegia by refractory hypotension and the need for any dose of continuous vasopressor infusion to maintain MAP over 65 mmHg in the setting of a CI of 2.2 L/min/m² and of a central venous oxygen saturation >60%. The patients were randomized to receive either 1,500 mg of vitamin C administered every six hours or normal saline (100-mL bag) in the placebo group. The study drug was administered over one hour. Of 456 patients, 52 (11.4%) were eligible, and 50 (11.0%) were included. In this setting, the administration of high-dose vitamin C did not lead to a faster resolution of PV or a decrease in the total norepinephrine equivalent dose administered [39].

Inhibitors of NOS

MB: MB has been explored as a treatment for vasoplegic states following cardiac surgery, a condition affecting up to 20% of patients and associated with increased costs and poor outcomes. First studied in the 1990s, MB has gained renewed interest despite some controversy over its use [40,41].

MB is considered safe and effective when given in recommended doses for vasoplegia. It does not cause endothelial dysfunction and is especially effective in cases involving NO upregulation. Unlike vasoconstrictors, MB works by inhibiting NOS and guanylate cyclase, reducing cGMP accumulation and restoring vascular tone. Its application has been explored in various clinical settings, including liver transplantation, sepsis, and neurogenic shock. The typical dosage is 2 mg/kg IV bolus, followed by a continuous infusion, as plasma levels decline rapidly within 40 minutes [42].

Prophylactic or therapeutic use of MB for vasoplegia?: Most studies on MB for vasoplegia are small case series, showing reduced vasopressor doses and improved outcomes, but they do not support its routine use after bypass surgery. Levin et al. found that MB (1.5 mg/kg) shortened vasopressor use and had no mortality, compared to six deaths in the control group [43]. Mazzeffi et al. observed that 44.3% of patients treated with a 2 mg/kg bolus had decreased norepinephrine use and 8.3% mortality, while non-responders had a 20.4% mortality rate [44]. Weiner et al. found worse outcomes with MB after matching for propensity scores [45].

The prophylactic use of MB in high-risk patients scheduled for cardiac surgery under bypass to mitigate hypotension seems interesting. However, it is essential to carefully weigh the potential risks against the benefits of this approach. In a relatively small and older study, Maslow et al. randomized 30 patients taking ACE inhibitors undergoing cardiac surgery to receive either MB (3 mg/kg) or saline after initiating CPB. All patients had a similar decrease in mean arterial blood pressure and SVR with the onset of CPB. Comparing the groups, MB increased MAP and SVR, and this effect lasted for 40 minutes. The saline group demonstrated a persistently reduced MAP and SVR throughout CPB and received more vasopressors and high levels of lactate. The authors conclude that MB could be indicated for treating hypotension during CPB for patients receiving preoperative ACE inhibitors [46].

In patients considered at high risk for vasoplegia (using ACE inhibitors, calcium channel blockers, and heparin), the MB administration before surgery reduced the incidence and severity of vasoplegic syndrome. These patients presented higher SVR and reduced norepinephrine use and intravascular fluid requirements [47].

A study conducted by Mehaffey et al. retrospectively examined the effects of early (in the operating room) versus late administration (in the ICU) of MB on patients who underwent cardiac surgery with bypass and had a higher incidence of comorbidities. Early administration of MB significantly improved survival rates and reduced the risk-adjusted rate of major adverse events in these patients [48].

Risks and toxicity of MB: MB is usually administered postoperatively as a single dose (1.5-2 mg/kg). It should be avoided in patients taking SSRIs, serotonin-norepinephrine reuptake inhibitors, or tricyclic antidepressants, as it can trigger serotonin syndrome, a life-threatening condition that includes altered mental status, coma, neuromuscular excitability, clonus, hyperreflexia, myoclonus, tremor, and pyramidal rigidity, and autonomic instability characterized by hyperthermia, tachycardia, diaphoresis, and mydriasis. MB also causes a blue-green urine color and may interfere with pulse oximetry. It should be used cautiously in patients with glucose-6-phosphate dehydrogenase deficiency due to the risk of hemolytic anemia. The half-life of MB is about 5.25 hours, and it is primarily excreted in urine [40,49].

Hydroxocobalamin

In the United States, hydroxocobalamin, a precursor of vitamin B12, is an approved treatment for cyanide intoxication due to toxic gas inhalation. Considering clinical use, there are several case reports on using hydroxocobalamin for PV in cardiac surgery, liver transplantation, and vascular surgery. Additionally, it is effective in treating vasoplegic syndrome and can be used alone or with MB. The medication works by reducing NO concentration and inhibiting its production, thus preventing severe hypotension, which moves its interest to treating vasoplegic shock. Most studies administered a 5-gram dose of hydroxocobalamin as a bolus over 10 or 15 minutes, with various reported protocols. However, the hydroxocobalamin dosage required to antagonize NO production during vasoplegic shock remains undefined. Hydroxocobalamin is considered safe; doses up to 30 grams have been reported to treat cyanide intoxication, although common side effects include chromaturia, erythema, and elevated blood pressure. While serious adverse effects such as anaphylaxis and acute renal injury are rare, they can occur [50].

The study by Seelhammer et al. focused on 12 patients who underwent various cardiac surgery procedures, receiving support from artificial heart mechanical devices. These patients exhibited criteria for vasoplegic shock. They were each given a single 5-gram dose of hydroxocobalamin over a median duration of six hours. As a result, all patients experienced a simultaneous decrease in norepinephrine levels lasting at least 600 minutes. The study did not determine the safety protocol and impact on survival. Although the mortality rate in this series is admittedly higher than that of other reports, the authors referred to hydroxocobalamin as a salvage intervention after the failure of conventional therapies [51].

MB or hydroxocobalamin for vasoplegic syndrome in cardiac surgery?: A study by Kram et al. compared the effectiveness of MB (1.2 mg/kg) and hydroxocobalamin (5.0 grams) in treating vasopressor-refractory vasoplegic syndrome after cardiac surgery. Both agents were equally effective in reducing vasopressor use [52]. In another study by Feih et al., a combination of MB and hydroxocobalamin showed potential benefits over MB monotherapy, as it allowed for lower doses of MB and possibly more significant reductions in vasopressor use, although the sample size was small [53].

## Conclusions

Patients undergoing cardiac surgery often experience a harmful inflammatory response that can worsen outcomes. This response is influenced by preoperative factors, age, and genetic differences, demonstrating significant redundancy and pleiotropism. Addressing this issue requires more than just targeting a single pathway. While synthetic vasopressors are commonly used to treat vasodilatory shock, a multimodal approach has been proposed, incorporating vasopressin, corticosteroids, angiotensin II, MB, and hydroxocobalamin. Given that vasopressor-refractory shock is associated with a mortality rate of 65-90%, there is growing interest in vasopressor-sparing strategies aimed at reducing the harmful effects of catecholamines and facilitating hemodynamic restoration.

In short, in the presence of refractory hypotension (MAP <65 mmHg with preserved contractility, norepinephrine >0.5 mcg/kg/min and/or vasopressin 0.06 IU/min after volume adjustment), consider introducing MB (two bolus doses of 2 mg/kg were followed by an infusion of 0.5 mg/kg/h for six hours) or hydroxocobalamin (a bolus of 5 g over 10-15 min). The association of both MB and hydroxocobalamin could also be considered.
